# Complementarity of Raman and Infrared spectroscopy for rapid characterization of fucoidan extracts

**DOI:** 10.1186/s13007-021-00830-6

**Published:** 2021-12-20

**Authors:** Signe H. Ptak, Lee Sanchez, Xavier Fretté, Dmitry Kurouski

**Affiliations:** 1 Department of Chemical Engineering, Biotechnology and Environmental Technology, University of Southern Denmark, Odense, Denmark; 2 Department of Biochemistry and Biophysics, Texas A&M University, College Station, USA

**Keywords:** Sulfated polysaccharides, Fucoidans, Raman spectroscopy, Infrared spectroscopy

## Abstract

**Background:**

Fucoidans are sulfated polysaccharides from the cell-wall of brown algae. They have a wide range of applications in medicine, including regenerative medicine, ophthalmology, cancer, and autoimmune disease. Biological activity of fucoidans directly depends on their structure, which remains poorly understood. This is primarily because the polymeric nature of these molecules limits the use of nuclear magnetic resonance and mass spectrometry, classical tools of structural biology for their structural characterization. Raman and Infrared spectroscopies are non-invasive and non-destructive techniques that can be used to probe the structural organization of biological specimens. In this study, we investigate the potential of Raman and Infrared spectroscopy for structural analysis of several fucoidan extracts.

**Results:**

Our results show that Infrared and Raman provide different but complimentary information about the structure of crude extracts of fucoidans, revealing the presence of minor impurities from co-extractants. We also found that at high extraction temperatures acidic conditions limit formation of melanoidins, while also yielding relatively high sulfate ester fucoidan. However, at high temperatures, water extraction may potentially result in formation of advanced glycation end products. Their presence could be problematic for fucoidan extracts intended for medicinal use, as advanced glycation end products have been linked to endocrine interruption mechanisms in vivo by crosslinking to and permanently altering extracellular matrix proteins.

**Conclusion:**

Raman and Infrared can be used as complementary tools for rapid screening of crude fucoidan extracts, which can be a valuable tool for assessing impurities that remain after extraction.

## Background

Sulfated polysaccharides from the cell-wall of brown algae (fucoidans) are a di- verse family of polydisperse hetero-polymers with a wide range of applications in medicine, including regenerative medicine, ophthalmology, cancer [1, 2], and autoim- mune diseases [3]. Their structure-pharmacological activity relationship has not been fully elucidated due to their structural heterogeneity and taxonomic variability. A growing body of evidence suggests that pharmacological activity of fucoidans also depends on the extraction methods using for their processing.

Fucoidan extraction typically involves soaking the seaweeds in aqueous or acidic solutions at ambient or high temperatures, while isolation is often performed by several recipitation steps involving calcium chloride and ethanol to desalt and remove low-molecular weight compounds. Other methods include the use of enzymes [4], microwave-assisted extraction [5, 6], and membrane filtration [7, 8]. Extraction and purification are closely linked to the structure and bioactivity of a fucoidan. Fucoidan may undergo structural changes during extraction, which affects their bioactivity. Microwave-assisted extraction provides rapid extraction of fucoidan, generating high yields and eliminating further extraction steps. The drawback of this method is thermal degradation of the backbone of the native fucoidan polymer: microwaving at temperatures below 100 °C provides high yields and fucoidans high in fucose, but the fucose content decreases with increasing temperature, resulting in fucoidans with glucuronic acid as the main monosaccharides [6]. The fucoidan backbone may carry both carbohydrate (mostly l-fucopyranose and d-glucuronic acid) and non-carbohydrate substituents (sulfate and acetyl groups) [9]. Other monosaccharides may also be part of the backbone and/or part of the polymer branches in some algae species. Nearly all structural studies on fucoidan focus on elucidation of the relationship between bioactivity and the degree of sulfation. At the same time, more complex structural analysis of these biopolymers is required to fully reveal their biological activity. For instance, structural features like branching and molecular weight reportedly also affect the bioactivity. Cho et al, noted that a fucoidan fraction of 5–30 kDa exhibited a higher inhibitory effect on tumor growth compared to a fraction with a molecular weight of >30 kDa, regardless of the higher molecular weight fucoidan having a higher sulfate content [2]. Sulfate esters do have a noticeable effect on several properties, including physicochemical ones. Wei et al, found that decreasing the amount of ester sulfate groups in *L. Japonica* fucoidan increased aggregate formation, due to an abundance of inter-chain hydrogen bonds [10]. Such conformational changes are less studied, even though they may affect polymer activity *in vivo*. One key factor is the presence (or absence) of side chains throughout the polymer, as they reduce the flexibility of the oligosaccharide back- bone and stiffen the polymer. Increased stiffness may be favorable in some cases, as this forces the fucoidan polymer to adopt another conformation. One fucoidan polymer with several side chains was reportedly adopting a conformation recognized by certain receptor proteins [11]. Highly flexible, linear polymers, on the other hand, can axially rotate each bond in the polymer chain to fit the whole fucoidan molecule to a certain steric arrangement. This structural property enables the fucoidan to interact with positively charged amino acid residues of receptor proteins [12]. Having such massive impact on bioactivity, it is very important to determine the fucoidan structure after extraction.

Structural analysis of crude extracts directly after extraction may reveal what fucoidan features (if any) are altered by the extraction method. Ideally, this should be a rapid analysis, performed in real-time. Infrared and Raman are versatile, non-destructive tools for the identification of biomolecules in plant cells and tissues. These techniques are highly advantageous during production processes, as they can be used with minimal preparation for in-line analysis. Using both Raman and FT-IR provides structural insight that would otherwise be unattainable from one technique alone. Bond vibrations with strong intensities in the IR spectra are typically weak contributors to Raman spectra and vice versa. For an IR transition, the vibrational motion is accompanied by a change in dipole moment, while it is the change in polarizability of the electron cloud within the molecule that lead to strong Raman bands [13]. This paper explores the complementarity of Raman and FT-IR structure analysis on fucoidan extracts.

## Results and discussion

### Size-exclusion chromatography

Table 1 shows the proposed size distribution of each fucoidan extract and the two structural references. Two acid extracts show varying degrees of hydrolysis, with a main molecular weight of 1545 kDa and 961 kDa for the sulfuric acid and hydrochloric acid extract, respectively. The water extract contains the most diverse size composition, with a main molecular weight of 470 kDa. A massive poly- mer with a molecular weight above 50000 kDa accounts for 16% of the water extracts, which greatly exceeds the average weight of fucoidan. This suggests that polymerization or the creation of another compound has taken place during fucoidan extraction.

**Table 1:** Chemical characterization of fucoidan samples and references

Sample	Mw [kDa]	Mw Relative intensity [%]	Sulfate ester content [%]	Nitrogen content
Fucoidan reference	1884	91.15	31.56 ± 2.44	0.02 ± 0.02
	809	2.8		
	611	4.93		
	4	1.12		
Laminarin reference	5400	3.29	10.92 ± 2.44	0.16 ± 0.02
	3155	2.4		
	1010	9.8		
	598	0.62		
	12	1.21		
	7	82.68		
H_2_O extract	52,658	16.12	16.2 ± 2.44	0.23 ± 0.02
	3260	26.66		
	470	34.58		
	340	8.78		
	9	3.34		
	7	5.84		
	5	3.42		
	4	1.27		
H_2_SO_4_ extract	1545	88.48	17.85 ± 2.44	0.12 ± 0.22
	611	5.34		
	8	2.31		
	7	3.15		
	5	0.72		
HCl extract	961	84.67	20.93 ± 2.44	0.16 ± 0.02
	547	7.34		
	8	4.31		
	7	3.09		
	5	0.59		

Size distribution and the intensity of each polymer weight in RID, and the results of the elemental analysis on the sulfate ester content (calculated from S%) and the nitrogen content of each extract and reference

### Elemental analysis

Table 1 shows the sulfate ester and nitrogen content of each fucoidan extract and reference. The nitrogen content is typically quite low for brown algae, as they do not contain significant amounts of protein.

### Raman and infrared on references and extracts

**Fig. 1:**
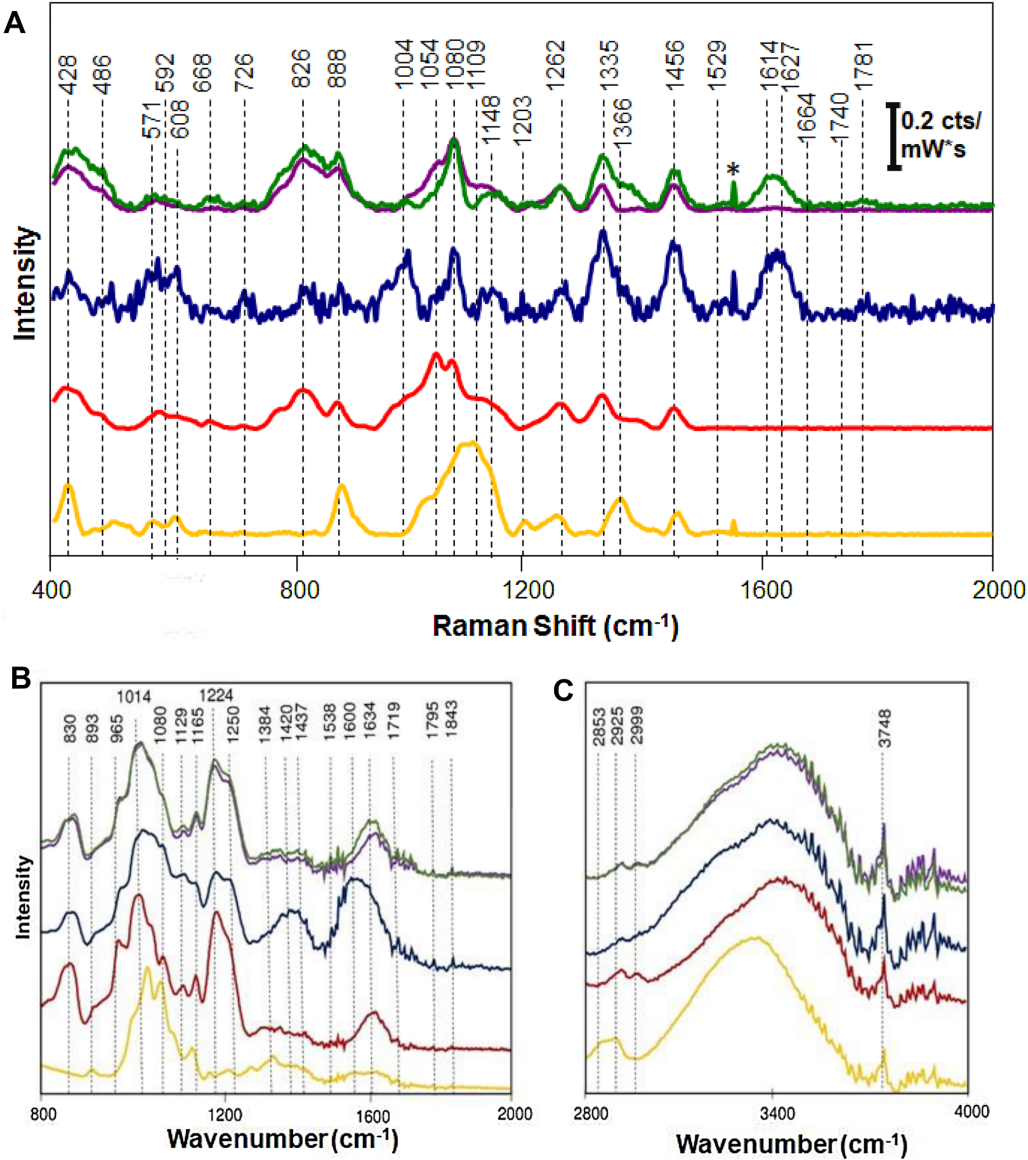
Raman (**A**) and infrared (**B** and **C**) spectra of fucoidan extracts. The purple spectrum indicates fucoidan extraction with 10 mM H_2_SO_4_, while green and blue is 100 mM HCl and pure water, respectively. Red shows the reference fucoidan and yellow the laminarin reference sample. The intensity of Raman spectrum is shown in counts (cts) divided over mW of the 830 nm laser power multiplied by spectral acquisition time

In the Raman and IR spectra collected from the fucoidan extracts (see Fig. 1, vibrational bands that can be assigned to carbohydrates, melanoidins and amides where observed. In the IR spectra, bands belonging to carbohydrates, melanoidins, sulfate esters, and amides (Table 2) were seen. The peak intensities among the extracts differ. This may be a result of pigmented compounds formed during mi- crowave extraction. The colourants may absorb a significant fraction of laser light.

**Table 2:** Vibrational bands in fucoidan and laminarin

RS	IR	Vibrational Mode	Assignment
428		C–O–C + C–C–C ring deformation	Carbohydrate [14]
486			
571		*δ*(C–C–O) + *τ* (C–O)	Carbohydrate [14]
592			
608		*δ*(C–C–O)	Carbohydrate [14]
668			
726		*δ*(C–C–O)	Carbohydrate [14]
826		C–O–S stretching, *α*-1.3 bond	Fucoidan
	830	C–O–S stretching	*α*-pyranose [15], C–O–S of
			eq. sulfate ester on C-2 [16]
888		*δ*(C–C–H) + *δ*(C–O–C)	Carbohydrate [17]
	893	C–O–S stretching	equatorial sulfate ester of a
			pyranoid sugar [18, 19]
	965	*ν*(C–O) in C–O–C linkage, *β*(C–O–H)	Carbohydrate [13]
1004		Aromatic ring breathing	Aromatic compound from
			Maillard reaction
	1014	*β*(C–O–H) + *ν*(C–C), C–O–S Stretching	Trisaccharide [13], fucoidan
1054		*ν*(C–O) + *ν*(C–C) + *δ*(C–O–H)	Carbohydrate [14]
1080	1080	*ν*(C–O) + *ν*(C–C) + *δ*(C–O–H)	Carbohydrate [13, 14]
1119		*ν*(C–O) + *ν*(C–C) + *δ*(C–O–H)	Carbohydrate [14]
	1129	C–O–C + C–O–S stretching hfill	Fucoidan
1148		*ν*(C–O–C) + *ν*(C–C) in glycosidic linkages,	Carbohydrate [13]
		asymmetric ring breathing	
	1165	*ν*(C–O)	Glucose [13]
1203		*ν*(C–C) + *ν*(C–O)	Carbohydrate [14]
	1224	S–O stretching	Sulfate ester [17]
	1250	S–O stretching	Sulfate ester shoulder
1262		*δ*(C–C–H) + *δ*(C–C–H) + *δ*(C–O–H)	Carbohydrate [14]
1335		*ν*(C–O) + *δ*(C–O–H), *δ*(C–H)	Carbohydrate [14],
			Alginate [20]
1366		*δ*(C–H) stretch	MG alginate [20]
	1384	CH_3_ bending, symmetric	Carbohydrate [18]
	1420	C–H deformation	Melanoidin
	1437	O–H rocking	Monosaccharide
1456		*δ*(C–H) + *δ*(CH_2_) + *δ*(C–O–H), CH, CH_2_ (in plane) + C–O–H deformations + asymm	Carbohydrate [14], Alginate [20]
		COO-	
1529		–C=C– (in plane) + vasym COO-	Carotenoids [21],
			Alginate [20]
	1538	*ν*(O–C–N)	Amide II [22]
	1600	*δ*(C–C–H) + v(C=C)	Amide [23], Melanoidin [24]
1614		*ν* asym COO-	Alginate [20]
1627		*ν*(C–C) + v(C=O)	Amide [25], Melanoidin
	1634	*ν*(C=O) + *ν*(C=C) + *ν*(C=N)	Amide I [23], Melanoidin [24],
			unhydrated *β*-sheets [26, 27]
1664		*ν*(C=O) stretching	Acetylamide [28]
	2853	*ν*(CH_3_) stretch, symmetric	
	2925	v(CH_2_) stretch, symmetric	Carbohydrate [22]
	2999	C-H stretching	Carbohydrate [18]
	3400	O–H stretching	Carbohydrate [15]

### Infrared analysis of fucoidan extracts

A vibrational band at 830 cm^-1^ was observed in the IR spectrum of all fucoidan extracts and in the fucoidan standard. This vibrational band can be assigned to *α*- pyranose [17]. Since only one band is present in this linkage region, it is highly likely that this fucoidan has a simple, linear structure. It has been shown that *Fucus vesiculosus* fucoidan contains alternating *α*(1 *→* 3) and *α*(1 *→* 4) linkages of L- fucopyranose units [29]. This indeed confirms that the polymer has a simple, linear structure. Laminarin functions as a beta-glucan storage polymer in brown algae, which explains why the 830 cm^-1^ band for *α*-pyranose is absent in the laminarin spectra. This region (the pyranose region) has previously been used to assign the positions of the sulfate ester groups (-OSO_3_-) on the monosaccharide units [30]. Extracting such structural information from the IR alone can be a powerful tool when characterizing the structure and potential function of a fucoidan extract. The -OSO_3_- position influence polymer properties, and axial positions greatly determine the conformational flexibility of the fucoidan polymer [31]. Two bands in the pyranose region can be used to uncover the -OSO_3_- substitution pattern; a strong band at 844 cm^*−*^1 denotes axial position, while a shoulder band at 820 cm^-1^ shows an equatorial substitution [30]. These bands can also be assigned to the C–O–S, C–O, C–C and S–O vibrations [32], which makes unambiguous assignment of these bands challenging.

From the recorded infrared spectra, vibrational bands for sulphated polysaccha- rides were observed at 830 cm^-1^, 893 cm^-1^, 1014 cm^-1^, and 1129 cm^-1^. Although the bands seem to shift somewhat depending on the extraction method, they can be assigned to the C–O–S vibration of a -OSO_3_-. The 830 cm^-1^ band was found to be a distinguishing feature of fucoidan in the spectra as it shows (1) the molecule is sulfated, as given by the C–O–S vibration from -OSO_3_- (possibly on C-235) and (2) the joined monosaccharides have *α*-conformation. Although we did not observe a band for an equatorial substitution pattern at 820 cm^-1^, we observed a band at 893 cm^-1^ in IR, which can be assigned to the general C–O–S stretching of an equatorial-OSO_3_- of a pyranoid sugar [18]. A C–O–S band at 893 cm^-1^ was also observed for laminarin and the elemental analysis confirms that the laminarin standard is in- deed sulfated, albeit to a lesser degree than the fucoidan extracts and the fucoidan standard in this study. The band at 1224 cm^-1^ and the shoulder at 1250 cm^-1^ is often used for detection of sulfated polysaccharides and they can be assigned to S–O stretches from sulfate esters. These bands are seen in all the IR spectra collected from fucoidan, while they are absent in the spectra collected from laminarin. These bands are stronger in the spectra of acid extracts and in the Sigma standard, suggesting that at high extraction temperatures, the amount of sulphated fucoidan is larger in acid-based extracts. The peak intensities and shape of the 1014 cm^-1^ and the 1224 cm^-1^ in the water extracts also suggests that some of the fucose has been lost during extraction. Lowering the pH may preserve the sulfate ester groups at elevated extraction temperatures. This preserving effect may be due to the mitigation of the Maillard reaction (reaction between a reducing sugar, such as glucose, and the carbonyl group of free amino acid), as lowering the pH reduces the reaction rate. Although the calculated sulfate ester content in laminarin may be from not dialyzing the extract after extraction, the absence of the 1224 cm^-1^ and the 1250 cm^-1^ shoulder bands from the laminarin spectra may suggest that, as the degree of sulfation increases, not only does the intensity of the sulfation bands increase, more sulfation bands can be seen in the IR spectra.

Vibrational bands at 1600 cm^-1^ and 1634 cm^-1^ can be assigned to melanoidins. In IR, this region may denote C=C, C=O, and *α*,*β*-diketones [24]. We propose that melanoidins were formed during extraction as a result from the Maillard reaction. The intensity of the 1600 cm^*−*^1 and 1634 cm^*−*^1 bands are higher in the water extracts, which we attribute to the pH of the extraction solvent. The acid extracts had lower pH during extraction, which may mitigate the Maillard reaction and result in less Maillard reaction products.

The vibrations 1420 cm cm^-1^ and 1634 cm^-1^ are also highly solvent (or pH) dependent, as the intensity is much higher in the water extracts. The 1420 cm^-1^ band can be assigned to –C–H deformation, while the unambiguous assignment of the 1634 cm^-1^ band is more challenging. This band is often assigned to the amide I, but it may also denote a *β*-sheet structure for protein and to C=C stretching and even to C=N stretching vibrations for melanoidins [33, 34]. Based on the IR spectra and the elemental analysis, we propose that the acid extracts contain low amounts of protein, while the water extracts likely contains both a small amount of protein and the brown, nitrogenous, high molecular weight polymers, melanoidins. Structural information on melanoidins is limited due to their high structural complexity and diversity, but they are widely found in processed foods. Two minor peaks at 2925 cm^-1^ and 2994 cm^-1^ were also observed. These peaks are likely *δ*(CH2) deformations, al-though these peaks are normally more prominent in carbohydrates. This peak is more pronounced in the laminarin spectra. This intensity difference is related to the monosaccharide composition of the laminarin and the fucoidan polymer. Unlike glucose, fucose does not contain a hydroymethyl group (–CH2–OH), only a methyl group (–CH3). The small, shifted peaks observed in the fucoidan extracts could po- tentially be from laminarin contamination in the fucoidan extracts. The presence of laminarin was confirmed for all extracts by the 1165 cm^-1^ band, which we assign to the *ν*(C–O) stretch from glucose.

Raman analysis of fucoidan extracts Raman spectrum of the water extracts exhibits poor signal to noise ratio, likely as a result of melanoidins and potentially other Maillard reaction products. At the first stage of the Maillard reaction, Amadori products are formed. As the Maillard reaction advances, these products may cross-link with adjacent proteins (or other amino groups), creating fluorescent polymeric aggregates, also called advanced glycation end products [35, 36] and simultaneously producing melanoidins.

Prolonged heating of the extracts is undesirable, as it may result in samples that are unsuitable for Raman spectroscopy. Melanoidins can be converted into fluorescent nanoparticles during heating, which could negatively affect the Raman Scattering [37]. Two wavebands, 1004 cm^-1^ and 1627 cm^-1^, were very distinct and could hint at the presence of advanced glycation end products in the water extracts. It has been reported that the 1004 cm cm^-1^ band is ring breathing of the amino acid phenylalanine [25, 38], however, glutamic acid (present as glutamate) and aspartic acid make up a noteworthy portion of the total amino acid content in brown algae [39]. One can expect that this peak is due to the ring breathing of an advanced glycation end products. The band at 1627 cm^*−*^1 can be assigned to v(C=O), which is characteristic for amides, and to v(C=C). We hypothesized that this band can be assigned to melanoidins. Based on the Raman spectra and the size-exclusion chromatography results, it is highly likely that melanoidins are present, however, the presence of advanced glycation end products could not be definitively confirmed based on the collected data.

The differences between the two acid-based extracts is most apparent in the Ra- man spectra. These two extracts differ in the wavebands 1054 cm^-1^, 1148 cm^-1^, 1335 cm^-1^, 1366 cm^-1^, and 1627 cm^-1^. These bands are related to carbohydrate stretches. The band at 1054 cm^-1^ originates from *ν*(C–O), *ν*(C–C) and *δ*(C–O–H), while the band at 1148 cm^-1^ is from the stretching of *ν*(C–O–C) and *ν*(C–C) in glycosidic linkages and asymmetric ring breathing. The band 1335 cm^-1^ is from the stretching and bending of *ν*(C–O) and *δ*(C–O–H). The band intensities for these stretches are higher for the extract prepared from hydrochloric acid, which indicates that this extract contains more free sugars than the other acid-based extract. The polysaccharides are hydrolyzed to a greater extend when extracted using a traditional extraction solvent. We found four bands that may be indicative of alginate and the intensity of these bands are higher in the hydrochloric acid extract. During extraction, the alginate was likely hydrolyzed and released into the extraction solvent. Gelling of the alginate by addition of calcium chloride solution was insufficient in its removal from the extract, possibly because the alginate fragments in solution had varying gelling properties and could not gel effectively.

We did not find any wavebands related to the S=O stretch in Raman, but we assigned the 826 cm^-1^ band to a C–O–S stretch. This peak, along with the 1224 cm^-1^ peak in infrared, is distorted in the water extracts. This could indicate that the water extract has less sulfate esters. This was also confirmed by the elemental analysis, although the difference in sulfate ester content is relatively small. The Sigma standard has a higher sulfate ester content, likely because some of the ester bonds were cleaved during microwaving. Shorter extraction times and lower temperatures conserve the sulfate esters more. The heat stability of the esters is quite high, how- ever, as evidenced in their high occurrence within the acid-based extracts. Sulfation is often reported as a main factor in bioactivity of fucoidans and rapid screening for determination of sulfate esters is highly needed. Raman and IR may provide more rapid screening than elemental analysis, as it measures the S=O stretch directly, but since these stretches are also within the region of carbohydrate stretches, it is difficult to make the analysis quantitative. It can be expected that polymer size also affects the position of the S=O peaks somewhat. The vibrations of the polymer be- low 1000 cm^-1^ depend on all monomeric units, as such, chain length may influence the spectra and may cause the S=O peaks to shift.

The question to ask is whether the structure and composition of fucoidans can be probed directly in algae without their extraction. To answer this question, we used atomic force microscopy Infrared (AFM-IR) spectroscopy. In AFM-IR, the sample surface is illuminated by pulsed tunable IR light that causes thermal expansions of the sample. These thermal expansions are recorded by a metalized AFM tip and converted to IR spectra. The probing depth of AFM-IR is within 100–300 nm, which allows for probing chemical composition of deeper laying material in the sample.

**Fig. 2:**
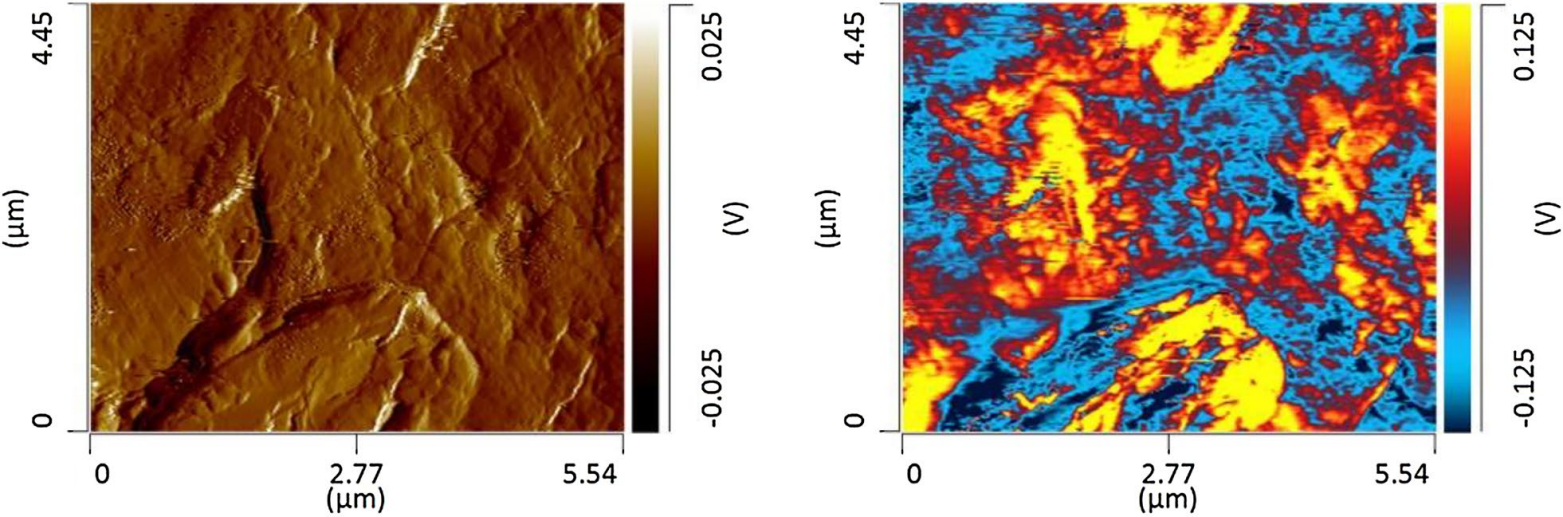
AFM (left) and corresponding AFM-IR (right) maps of the algae (*Fucus vesiculosus*) frond surface showing topology of the frond (AFM) and distribution of fucoidan (1170 cm^*−*1^) in the frond Nanoscale analysis of fucoidans directly in algae

We used AFM-IR to probe distribution of fucoidan in the intact algae (*Fucus vesiculosus*) frond (Fig. 2). For this, we measured change in intensity of the 1170 cm^-1^ band, which can be assigned to sulfate esters [17, 40], the C–O–C asym- metric stretch of polysaccharides [41], the C–C–O asymmetric stretch for phenols [42], C—O stretching of sodium alginate [43], and the polysaccharide backbone [44]. Our results show uneven distribution of fucoidan in the frond with clearly localized island-structures. These findings suggest that AFM-IR may be used for non-invasive screening of potential seaweed candidates for polysaccharide extraction.

## Conclusion

In this paper we investigated the complementarity of Raman and IR spectroscopy to determine the effect of extraction solvents on fucoidan polymer structure. Our results show that Raman and IR provide different but complimentary information about crude extracts, particularly revealing how minor impurities from co- extractants can be detected without needing destructive and time-consuming analytical techniques. From the IR and Raman spectra, we found that, at high extraction temperatures, acidic conditions limit the formation of melanoidins while also yielding relatively high sulfate ester fucoidan. However, at high temperatures, water extraction may potentially result in the formation of advanced glycation end products, which could be problematic for fucoidan extracts intended for medicinal use, as they have been linked to endocrine interruption mechanisms *in vivo* by crosslinking to and permanently altering extracellular matrix proteins [39].

## Materials and methods

### Extraction of fucoidan from *Fucus vesiculosus*

For this study, three fucoidan polymers were extracted from *Fucus vesiculosus* (Bio- cean, Roscoff, France) using microwave-assisted extraction. The extraction proce- dure was adapted and modified for microwave-assisted extraction from the method provided by Fletcher et al. [45]. Extraction was carried out by microwaving at 120 °C for 30 min, using demineralized water, 10 mM H2SO4, and 100 mM HCl. Once cooled, the extracts were neutralized with 1 M NaOH. For alginate pre- cipitation, a solution of 35% calcium chloride was added to each extract for a total concentration of 1%. The extracts were then centrifuged (4 °C, 30 min) and the supernatant was recovered. Ethanol was added for a concentration of 40% v/v ethanol to precipitate laminarin. The extracts were centrifuged, and the supernatant was recovered. Ethanol was added to give a final concentration of 70% v/v ethanol to precipitate crude fucoidan. The extracts were centrifuged and the crude fucoidan pellet was washed with ethanol and acetone and left to dry to a constant weight. The crude Fucoidan extracts were solubilized in water, dialyzed and lyophilized prior to Raman and IR analysis. For more details, see reference [46]. A fucoidan and a laminarin standard (Sigma Aldrich) were solubilized in demineralized water and used as structural references.

### Size-exclusion chromatography

A standard curve for determination of the molecular weight was prepared by solubilizing pullulan standards (Shodex, Japan) with varying molecular weights (5, 10, 20, 50, 100, 200, 400, 800 kDa). The pullulan was then filtered and placed into HPLC vials. Size exclusion chromatography was performed on an Ultimate 3000 (Thermofisher, USA) with a refractive index detector. Separation of the standards and samples was achieved on an Agilent BioSEC 3 column (4.5 x 300 mm, 300 ^˚^A, 3 *µ*m dp).

### Elemental analysis

5 mg of sample was placed into a tin capsule using a pair of tweezers. Using a flat-tipped tweezer, the tin capsule was carefully folded into an airtight, flat square, which was transferred to the sample carrousel of a vario MACRO CUBE (Elementar, Germany) for combustion. The S% results from the elemental analysis was used to calculate the sulfate ester content via Eq. 1

1 $$ {\text{Sulfate\,ester}}\left( \% \right) = S\% \cdot{3}.{22} $$

### Raman and Infrared spectroscopy

Raman spectra of the extracts were collected using a hand-held Resolve Agilent spectrometer equipped with 831 nm laser source. The following experimental parameters were used for all collected spectra: 1s acquisition time, 495 mW power. Spectral background was corrected using iterative polynomial smoothing method. FT-IR spectra were acquired on Perkin Elmer 100 spectrometer equipped with attenuated total reflectance (ATR) module. For each reported spectrum, 15 spectra were recorded with a resolution of 4 cm^-1^ in the range of 4000–560 cm cm^-1^. A background spectrum was acquired immediately before the measurement. Both IR and Rama n spectra shown in the manuscript are raw spectra without any smoothing or pre-processing.

### Screening of seaweed surface by AFM-IR

AFM-IR imaging was conducted using a Nano-IR3 system (Bruker, Santa Barbara, USA). The IR source was a QCL laser. Contact mode AFM tips (Anasys Instruments Inc., Santa Barbara, USA) with a resonance frequency of 13 ± 4 kHz and a spring constant of 0.007–0.4 N/m were used to obtain all spectra and maps.

## Data Availability

All datasets for the current study are available from the corresponding author upon request.
